# Understanding the Structure, Thermal, Pasting, and Rheological Properties of Potato and Pea Starches Affected by Annealing Using Plasma-Activated Water

**DOI:** 10.3389/fnut.2022.842662

**Published:** 2022-02-07

**Authors:** Yizhe Yan, Baixiang Peng, Bin Niu, Xiaolong Ji, Yuan He, Miaomiao Shi

**Affiliations:** ^1^Henan Key Laboratory of Cold Chain Food Quality and Safety Control, College of Food and Bioengineering, Zhengzhou University of Light Industry, Zhengzhou, China; ^2^College of Food Science and Technology, Henan Agricultural University, Zhengzhou, China

**Keywords:** plasma-activated water, annealing, structure, physicochemical properties, potato starch, pea starch

## Abstract

In this research, annealing (ANN) using plasma-activated water (PAW) was first employed to modify potato and pea starches. Compared with the conventional ANN using distilled water (DW), the granular morphology of two starches was not significantly affected by PAW-ANN. The results of X-ray diffraction (XRD) and Fourier transform infrared (FTIR) spectroscopy showed that PAW-ANN could reduce the long and short-range ordered structure of potato starch while improving the long and short-range ordered structure of pea starch. Differential scanning calorimetry (DSC) analysis indicated that PAW-ANN lowered the gelatinization enthalpy of potato starch and increased the gelatinization enthalpy of pea starch. The analysis of viscosity and dynamic rheological characteristics illustrated that PAW-ANN reduced the peak viscosity and improved the gel strength of starch pastes. PAW-ANN represents a novel modification method for modifying the structure, reducing the viscosity, improving the gel strength of starch, and is very promising for applying in starch-based hydrogels and food additives.

## Introduction

Starch is a significant carbohydrate source for most organisms, which has been widely applied in the food industry, such as papermaking, adhesives and biodegradable plastics, sweeteners, pasta products, and some fermented products (1). Native starch has the limitations of poor hydration performance, low thermal stability, low shear resistance, and high retrogradation rate, while modified starch performs well in terms of compatibility and has a low retrogradation rate, pasty gelation tendency, and gel shrinkage (2). Therefore, modified starch is preferred in the food industry instead of native starch. The modification of starch usually has the following three methods, namely, physical, chemical, and enzyme modification (3). Among them, physical modification is relatively safe and low-cost, and can greatly reduce waste generation (4).

Annealing (ANN) is usually to treat starch for a period of time at a high moisture content (more than 40%, w/w) and a low temperature (below 60°C). As a green modification method, ANN has received extensive attention because it only involves water and heat (5). It can usually change the internal structure and physicochemical properties of starch while not damaging the integrity of granules, which makes starch easier to be processed and employed in certain specific circumstances. For example, it has been studied that annealed starch was more suitable for application in yogurt, sauce, and low-digestible foods than native starch (6).

Plasma-activated water (PAW) from plasma treatment of distilled water (DW) has the advantages of uniform function, green and environmental protection. PAW will generate an acidic environment and generate active substances, such as hydrogen peroxide, nitric acid, and peroxynitrite, which will cause changes in the pH, redox potential, and conductivity. Recently, PAW has been widely used in food and agriculture yields, such as promoting plant growth, preservation of seafood, and broad-spectrum sterilization (7). However, PAW was rarely applied in the starch modification. According to our preliminary studies, PAW alone had no significant influence on the structure and properties of the starch. Therefore, a combination of PAW and other modified technologies could be an effective strategy. Based on this assumption, we have recently explored the combination of PAW and heat-moisture treatment (HMT) on the structure and properties of maize starches (8). PAW-HMT provided an innovative strategy to regulate the structure and digestibility of starch. However, PAW-HMT effect remained limited compared with the conventional HMT (DW-HMT). Therefore, the development of new PAW combined modifications (such as PAW-ANN and PAW-ultrasound) is desired.

In this research, PAW was first employed during ANN to modify potato and pea starches. After being modified with PAW-ANN, the changes of structure and properties of potato and pea starches were investigated. It is worth mentioning that this green modification method is only involved in plasma, water, and heat. This research will not only propose a new way of starch ANN treatment, but also further expand the application of PAW in starch modification.

## Materials and Methods

### Materials

Native potato starch (13.2% moisture and 21.6% amylose) was obtained from Qinghai Weston Potato Industry Group Co., Ltd. (Xining, China). Native pea starch (11.3% moisture and 30.8% amylose) was obtained from Henan Enmiao Food Co., Ltd. (Zhengzhou, China). A hydrogen peroxide quantitative kit was obtained from Shanghai Sangon Biotech Co., Ltd. (Shanghai, China). Nitrite and nitrate determination kits were obtained from Beyotime Biotechnology Institute (Nantong, China). Other reagents used in this experiment were of analytical grade.

### Preparation and Characterization of PAW

The PAW was obtained by treating 100 ml of distilled water through plasma processing equipment (Easton Geake Automation Equipment Co., Ltd., Shenzhen, China) for 120 s. The power was 750 W and the working gas was compressed air (0.18 MPa) (8). The pH/oxidation-reduction potential (ORP) meter and conductivity meter (INESA Scientific Instrument Co., Ltd., Shanghai, China) were, respectively, employed for measuring the pH, ORP, and conductivity of the PAW. Additionally, the content of H_2_O_2_, NO^2−^, and NO^3−^ were determined by corresponding assay kits. Notably, PAW was suggested to be further used within 12 h.

### ANN of Potato and Pea Starches With PAW or DW

After drying at 45°C, the moisture content of potato and pea starches (30 g, dry basis) is about 5%. Then, the moisture content of them was adjusted to 60% using DW or PAW, respectively. The starch samples were put into the reactor with sufficient stirring and then heated in an air oven at 50°C for 12 h. Finally, modified starches were obtained through washing, drying, and sieving and marked as DW-Potato, DW-Pea, PAW-Potato, and PAW-Pea, respectively.

### Scanning Electron Microscopy

Morphology of native and modified starch granules was observed using a scanning electron microscope (JSM-6490LV, JEOL, Japan). A small amount of dry-based samples were adhered to the double-sided conductive adhesive and coated with gold in the ion sputtering device (Polaron Sputter Coat System, Model 5001, UK) for 120 s (8). The imaging acceleration voltage of Scanning electron microscopy (SEM) was 20 kV. A micrograph of representative particles was chosen and taken at a magnification of ×1,000.

### Polarization Light Microscopy

A suspension of starch samples (1%, w/w) was obtained by using a solvent of glycerol and water (1:1, v/v) and observed under polarized light microscopy (PLM) (BX53M, Olympus Co., Ltd., Japan). The images were observed and taken at ×200 for all starch samples.

### X-Ray Diffraction

Before analysis of X-ray diffraction (XRD), starch samples needed balancing water over a saturated NaCl solution at room temperature for 1 week (9). Starch samples were analyzed by using an X-ray diffractometer (D8 Advance, Bruker, Karlsruhe, Germany). The detailed test conditions and relative crystallinity (RC) calculation of the starch samples were based on our previous study (8).

### Fourier Transform Infrared Spectroscopy

Fourier transform infrared (FTIR) spectra of starch samples were determined by using an FTIR spectrometer (Vertex 70, Bruker, Karlsruhe, Germany). Before the test, potassium bromide should be dried at 105°C for 6 h. A certain amount of samples and potassium bromide (mass ratio: 1:100) were taken in an agate mortar, mixed, and grinded for about 2 min. The ground mixed samples were placed in a tableing mold and compressed with a tableting machine, where the pressure was maintained within the range of 10 MPa for about 1 min and then taken out for testing. Test conditions: scanning wave number 4,000–400 cm^−1^, resolution 4 cm^−1^, scanning time 64 s. The spectra were analyzed by OMNIC8.2 (Version 8.2, Thermo Nicolet Inc., Madison, WI, USA). The absorbance ratio at 1,047/1,022 cm^−1^ (R_1047/1022_) can be obtained by deconvolution with a peak width of 38 cm^−1^ and enhancement factor of 19 and employed to characterize the short-range ordered structure of starch molecules (8).

### Differential Scanning Calorimetry

Thermal properties of native and modified starches were obtained by using a differential scanning calorimeter (Q20, TA Instruments Inc., Newcastle, DE, USA). The starch (3 mg, dry basis) and distilled water were added with a microliter syringe to a total weight of 12 mg into an aluminum pan. The samples were equilibrated at room temperature for 12 h. Subsequently, the sample pans were gradually scanned from 30 to 120°C at a heating rate of 10°C/min (8).

### Rapid Viscosity Analyzer

Pasting characteristics of the starch samples were obtained by using a rapid viscosity analyzer (RVA4500, Perten Instruments, Hägersten, Sweden). Starch samples were suspended in distilled water to make a total weight of 28.0 g (8% dry starch, w/w) and then analyzed using RVA Standard Procedure 1 profile. The samples were equilibrated at 50°C for 1 min, heated to 95°C within 222 s, held at 95°C for 150 s, cooled back to 50°C for 228 s, and then equilibrated at 50°C for 2 min. During this process, the paddle speed was kept at 960 rpm for the first 10 s, and then kept at 160 rpm. The pasting curves and parameters were obtained by RVA system software.

### Dynamic Rheological Properties

The dynamic rheological properties of starch pastes from the RVA experiment were analyzed by using a rheometer (Discovery HR-1, TA Instruments Inc., Newcastle, DE, USA). Sample pastes were transferred to the rheometer plate (40 mm diameter and 1,000 μm gap) and equilibrated for 5 min at 25°C. The strain was set at 1% and the frequency was taken at 0.1–20 Hz. G′, G″, and tan δ (G″/G′) of samples were recorded.

### Statistical Analysis

All experimental data were recorded as the means ± SDs. ANOVA followed by *post-hoc* Duncan's multiple range tests (*p* < 0.05) was conducted to determine the significant differences between mean values using the SPSS 26.0 Statistical Software Program (SPSS Inc., Chicago, IL, USA).

## Results and Discussion

### Characterization of PAW

After 2 min of plasma treatment, the pH of PAW dropped rapidly from 6.4 to 2.72, the conductivity value increased from 4.55 to 764.67 μS/cm, and the ORP value increased from 247 to 569.67 mV (Table 1). The measured conductivity value can be used to detect whether there were active ions in the water, and the ORP can be used to reflect the macroscopic oxidation–reduction performance of all substances in the aqueous solution, depending on the concentration and strength of the oxidant in the solution. During the plasma discharge process, a large number of active chemical substances, such as reactive hydroxyl radicals, singlet oxygen, superoxide, ozone, and active molecule nitrogen species, were generated. These substances interacted with water molecules to further generate H^+^, H_2_O_2_, NO, NO^2−^, NO^3−^, ONOO^−^, and so on, which led to the changes in the pH value, conductivity value, and ORP value of the aqueous solution (10). In addition, after plasma treatment, the content of H_2_O_2_, NO^2−^, and NO^3−^ was increased from zero to 131.32, 1,905.22, and 2,075.17 μmol/L, respectively (Table 1), which further verified the above fact.

**Table 1:** Physicochemical properties and content of active substances of distilled water (DW) and plasma-activated water (PAW).

**Water**	**pH**	**Conductivity (μS/cm)**	**ORP (mV)**	**H_**2**_O_**2**_ (μmol/L)**	${\text{NO}}_{2}^{-}$(μmol/L)	${\text{NO}}_{3}^{-}$(μmol/L)
DW	6.40 ± 0.05^a^	4.55 ± 0.48^b^	247.00 ± 2.65^b^	ND	ND	ND
PAW	2.72 ± 0.01^b^	764.67 ± 1.53^a^	569.67 ± 1.53^a^	131.32 ± 0.91	1,905.22 ± 0.75	2,075.17 ± 2.69

*Values are means ± SD; means with the same letters in a column for the same starch do not differ significantly (p > 0.05). ND, Not detected*.

### Granular Morphology

#### Scanning Electron Microscopy

**Figure 1:**
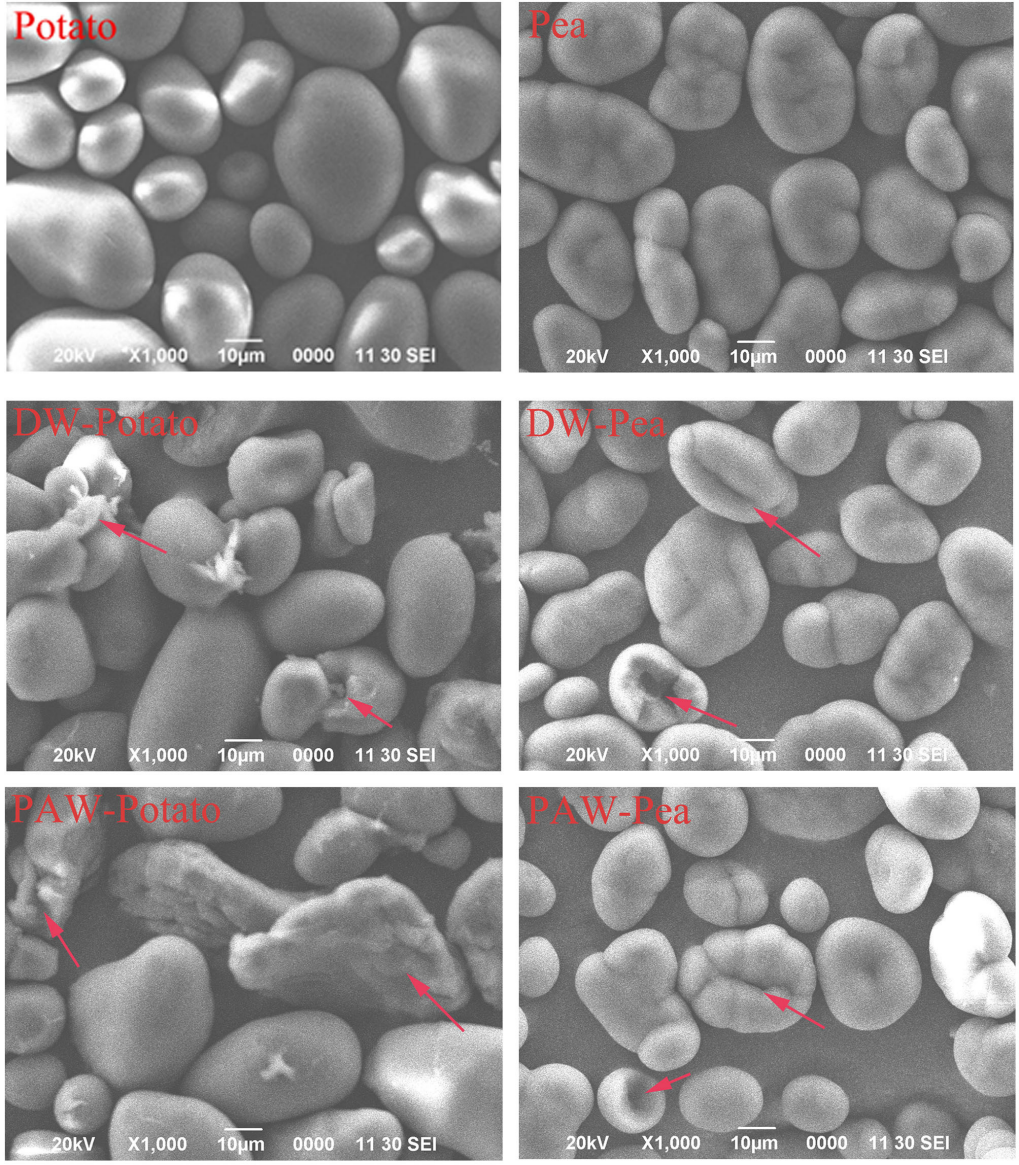
Scanning electron microscopy (SEM) images of native and modified starches.

The SEM micrographs of native and modified starch samples are displayed in Figure 1. Native potato starch granules were oval or spherical, with various sizes and smooth surfaces. The potato starch granules treated with DW-ANN and PAW-ANN had obvious dents and cracks. Native pea starch granules were slender, kidney-shaped, of different sizes, and had many folds (11). After DW-ANN and PAW-ANN, the shape of most pea starch granules did not change significantly, but the surface folds of the granules were deepened, and a few granules appeared with dents and cracks. However, PAW-ANN did not give significantly different results from DW-ANN for two starches.

#### Polarized Light Microscopy

**Figure 2:**
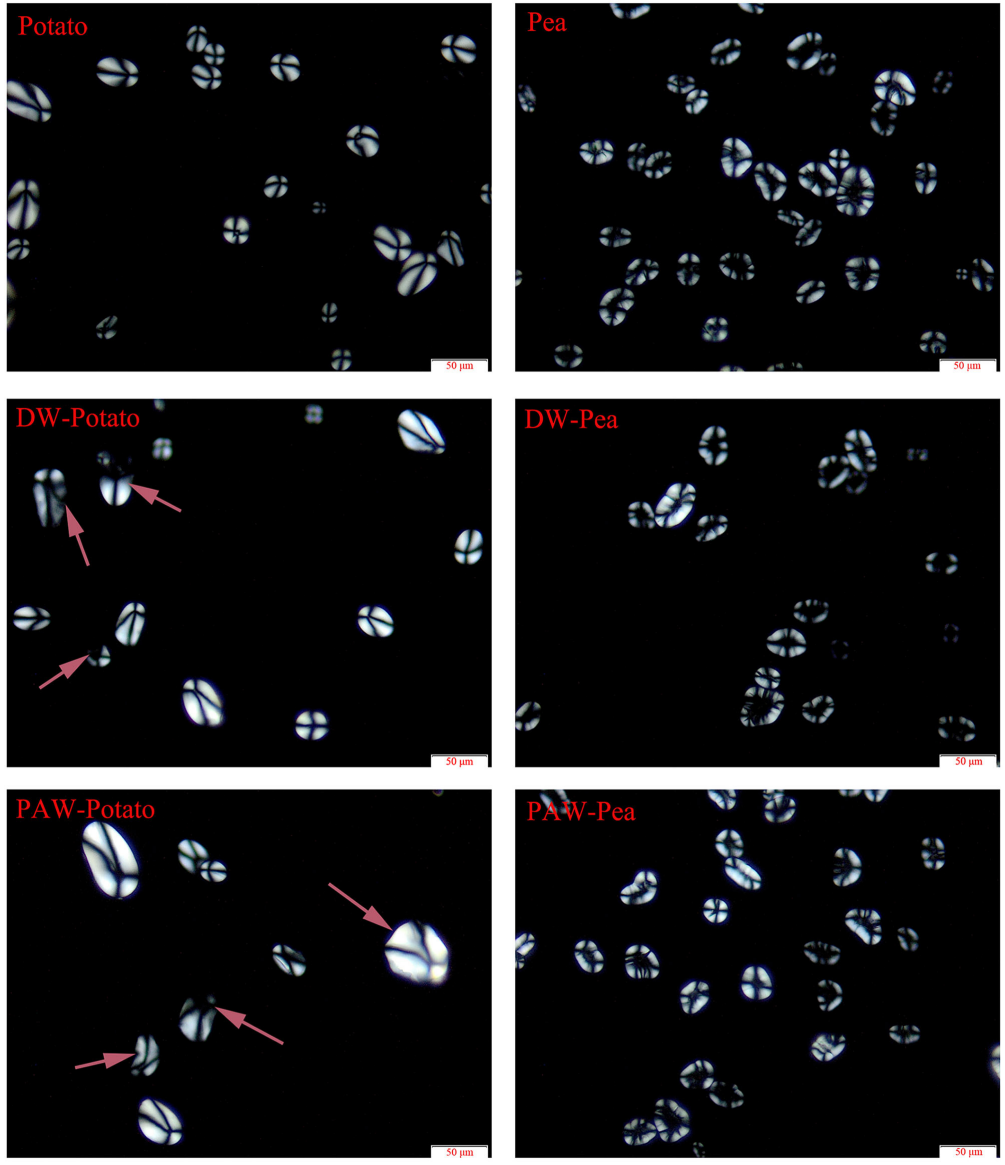
Polarized light microscopy (PLM) images of native and modified starches.

The PLM micrographs of native and modified starch samples are displayed in Figure 2. Native starch granules showed a characteristic birefringence pattern (Maltese cross). The strength of birefringence was related to the overall size, relative crystallinity, and crystallite orientation of the crystal grains (12). The polarized cross of native potato starch was complete and obvious. In contrast, the polarized cross of modified potato starch became fuzzy and the shape of the particles became irregular with cracks, which were similar to the observation by SEM. The difference in the polarized cross between native and modified pea starches was not obviously observed. Notably, there was no significant difference between PAW-ANN and DW-ANN for the two starches.

### Crystalline Structure

**Figure 3:**
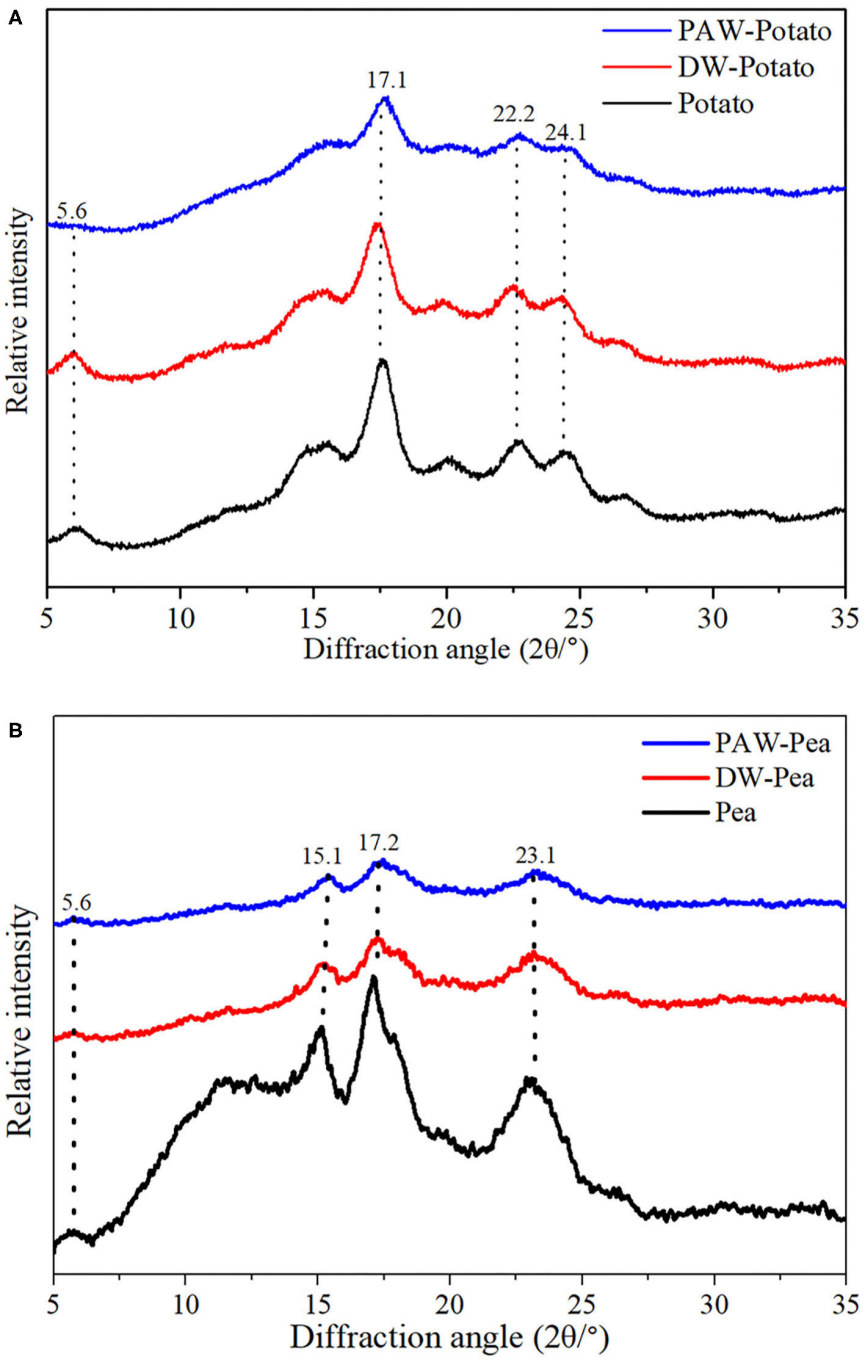
X-ray diffraction (XRD) patterns of native and modified starches. **(A)** Native, distilled water-annealing (DW-ANN) treated, and plasma-activated water-annealing (PAW-ANN) treated potato starch. **(B)** Native, DW-ANN treated, and PAW-ANN treated pea starch.

X-ray diffractograms of starch samples are displayed in Figure 3. Potato starch showed the characteristic diffraction peaks at *2*θ = 5.6, 17.1, 22.2, and 24.1 degrees, which was classified to the B-type structure (13). According to Table 2, the relative crystallinity (RC) of ANN-modified potato starch (DW-Potato and PAW-Potato) was lower compared with native potato starch. This might be because the double helix molecules of the B-type crystal structure were more sparsely arranged in space, forming a spiral cavity center (14), so ANN was more likely to destroy its crystal structure. This can be confirmed from the observations of the SEM and PLM of DW-Potato and PAW-Potato. In addition, the RC of PAW-Potato was lower than that of DW-Potato, which may be attributed to the acidic content of PAW. ANN could increase the sensitivity of potato starch to acid hydrolysis, resulting in its crystal structure being more easily hydrolyzed (15). The characteristic diffraction peaks of pea starch were mainly shown at *2*θ = 5.6, 15.1, 17.2, and 23.1°, which belonged to the diffraction of C-type starch (16). It can be concluded from Table 2 that the crystallinity of ANN-modified pea starch (DW-Pea and PAW-Pea) was higher than that of native pea starch. This might be because ANN made the initially weak or imperfect crystallites of pea starch gradually disappeared, while the remaining crystallites became more perfect due to melting and recrystallization (17). In addition, the RC of PAW-Pea was higher than that of DW-Pea, indicating that PAW can improve the RC of pea starch during ANN. This may be due to more hydrolysis of the amorphous area by the acidic components of PAW, resulting in a better orientation of the hydrolyzed starch crystallites (18).

**Table 2:** Long-range, short-range ordered structure, and thermal properties of native and modified starches.

**Samples**	**RC (%)**	**R_**1047/1022**_**	**T_**o**_ (**°**C)**	**T_**p**_ (**°**C)**	**T_**c**_ (**°**C)**	**ΔH (J/g)**
Potato	25.16 ± 0.35^a^	0.905 ± 0.003^a^	57.34 ± 0.24^c^	61.59 ± 0.23^b^	72.10 ± 0.67^b^	12.27 ± 0.43^a^
DW-Potato	22.13 ± 0.21^b^	0.862 ± 0.025^a^	62.32 ± 0.18^a^	66.32 ± 0.17^a^	74.33 ± 0.72^a^	9.39 ± 0.18^b^
PAW-Potato	15.20 ± 0.41^c^	0.783 ± 0.057^b^	61.86 ± 0.16^b^	66.04 ± 0.11^a^	73.32 ± 0.38^a^	7.31 ± 0.20^c^
Pea	25.40 ± 0.26^c^	0.877 ± 0.018^b^	57.92 ± 0.04^b^	65.07 ± 0.06^b^	75.58 ± 0.06^b^	6.69 ± 0.20^c^
DW-Pea	30.53 ± 0.31^b^	0.905 ± 0.011^ab^	66.97 ± 0.01^a^	69.74 ± 0.02^a^	76.25 ± 0.12^b^	8.50 ± 0.23^b^
PAW-Pea	34.93 ± 0.25^a^	0.934 ± 0.001^a^	66.92 ± 0.05^a^	69.76 ± 0.03^a^	78.42 ± 0.44^a^	9.66 ± 0.11^a^

*Values are means ± SD; means with the same letters in a column for the same starch do not differ significantly (p > 0.05)*.

### Short-Range Ordered Structure

**Figure 4:**
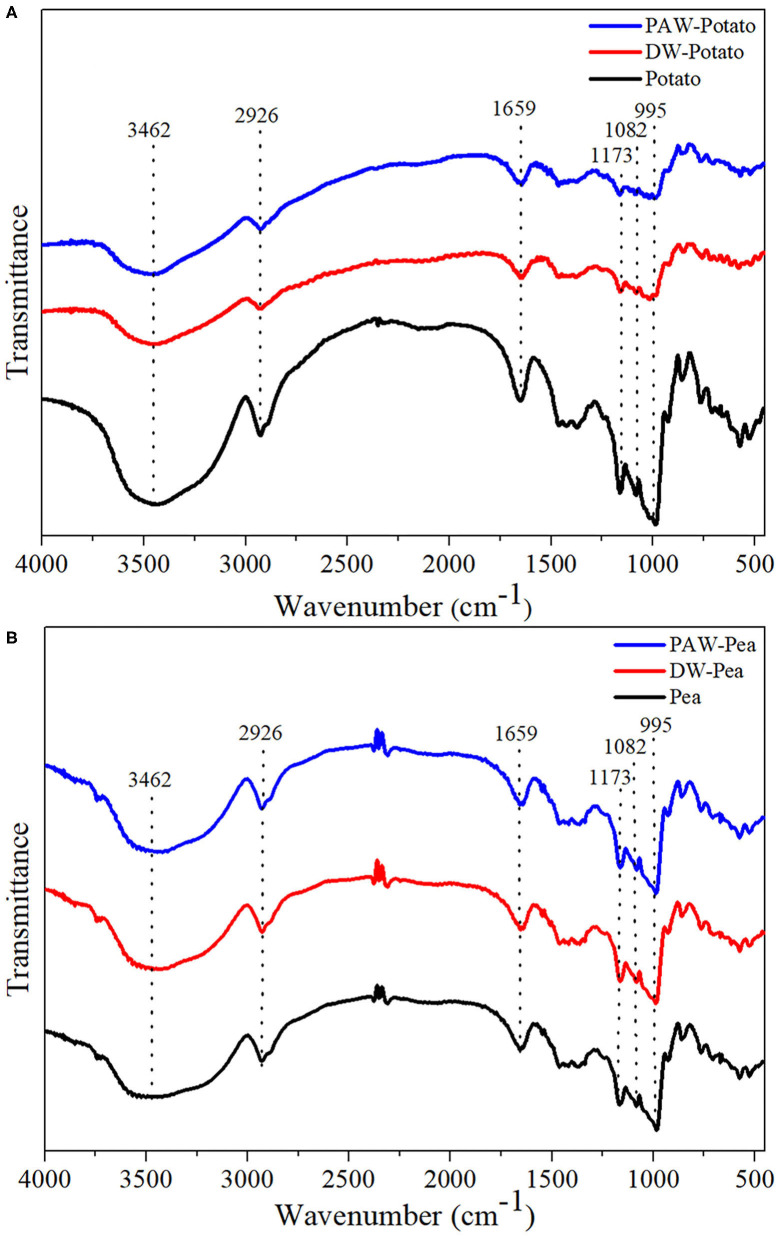
Fourier transform infrared (FTIR) spectra of native and modified starches. **(A)** Native, DW-ANN treated, and PAW-ANN treated potato starch. **(B)** Native, DW-ANN treated, and PAW-ANN treated pea starch.

The spectra of all starches showed similar trends, indicating that no new functional groups were formed (Figure 4). Studies have shown that the IR bands at 1,047 and 1,022 cm^−1^ were related to the ordered structure and amorphous structure of starch, respectively (19). The absorbance ratio of them (R_1047/1022_) can indicate the relative content of the short-range ordered structure of starch. R_1047/1022_ of native and modified starches followed the order: Potato > DW-Potato > PAW-Potato; Pea < DW-Pea < PAW-Pea (Table 2). For potato starch, R_1047/1022_ decreased successively because ANN caused starch granules to form granular pores, ruptures, and cracks, resulting in damage to the internal ordered structure. The presence of PAW further aggravated the destruction process. For pea starch, R_1047/1022_ increased sequentially, which might be due to the high moisture content and moderate heat energy existing in the ANN process that could produce more effective double helix stacking (6). Moreover, the acidic components of PAW could affect amorphous regions of starch and generate more short-chain amylose to form a new double helices structure. The result of R_1047/1022_ was in agreement with the result of XRD.

### Thermal Properties

**Figure 5:**
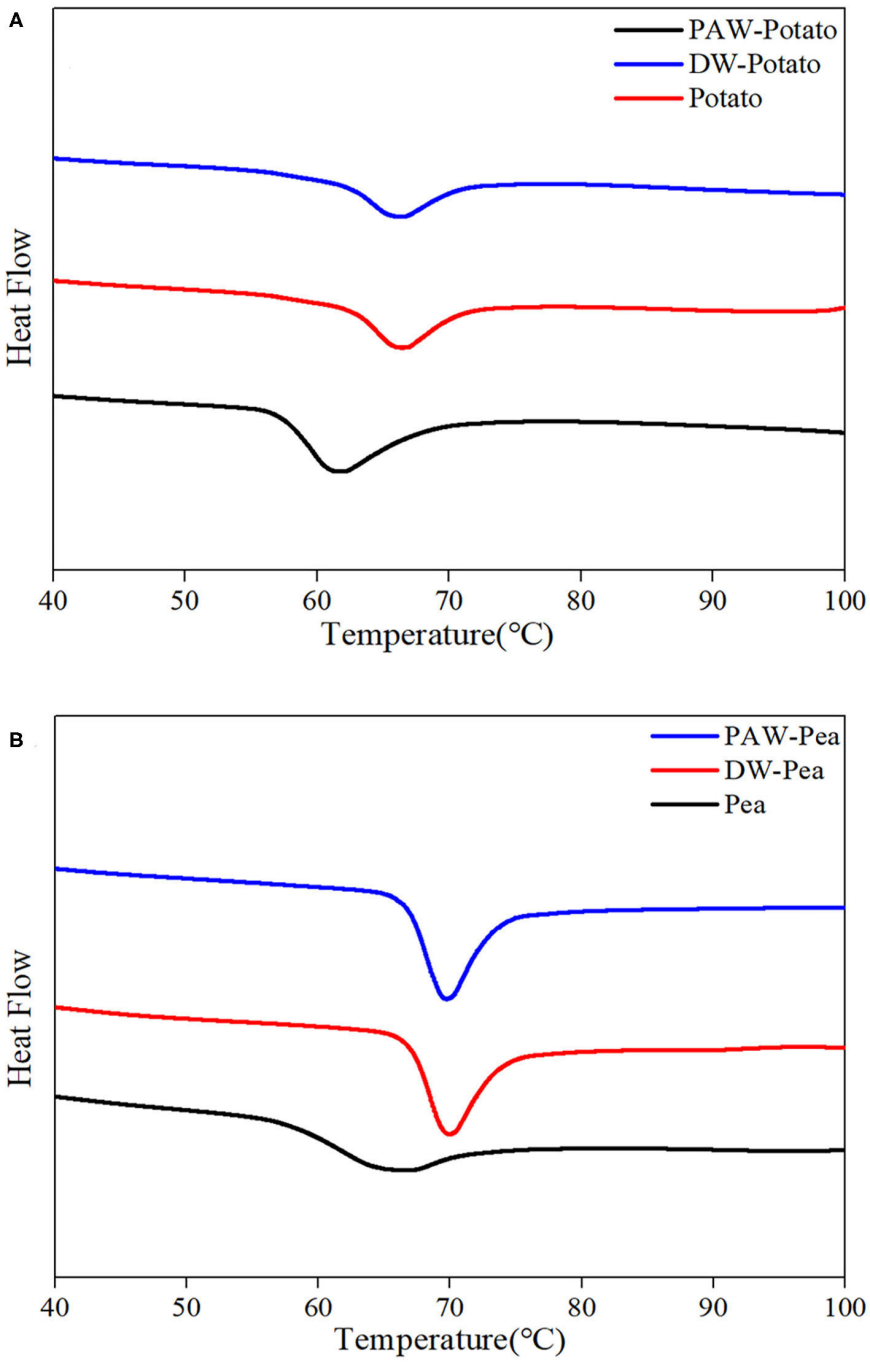
Thermal properties of native and modified starches. **(A)** Native, DW-ANN treated, and PAW-ANN treated potato starch. **(B)** Native, DW-ANN treated, and PAW-ANN treated pea starch.

The differential scanning calorimetry (DSC) curves of native and modified starches are shown in Figure 5. Table 2 presented the gelatinization transition temperatures (To, Tp, and Tc) and gelatinization enthalpy (ΔH). ANN resulted in a significant increase in the gelatinization temperatures of starch because ANN enhanced the interaction between amylose and amylose or amylopectin (20), inhibiting granule swelling and delaying gelatinization. However, the gelatinization temperatures of starches treated by DW-ANN and PAW-ANN were not significantly different. For potato starch, DW-ANN reduced the ΔH, and PAW-ANN can reduce the ΔH more, from 12.27 to 7.31 J/g. The decrease of the ΔH after ANN indicated the dissociation of unstable double helices in some starch granules (21), and the addition of PAW made this change more dramatic. For pea starch, DW-ANN increased the ΔH, and PAW-ANN can increase the ΔH more, from 6.69 to 9.66 J/g. The increase of the ΔH after ANN indicated more increase of effective double helix stacking in pea starch granules (22). After PAW-ANN, the increase of ΔH was due to the formation of new double helices, which was caused by the hydrolysis of amorphous regions (23). The ΔH result was in line with XRD and FTIR results.

### Pasting Properties

**Figure 6:**
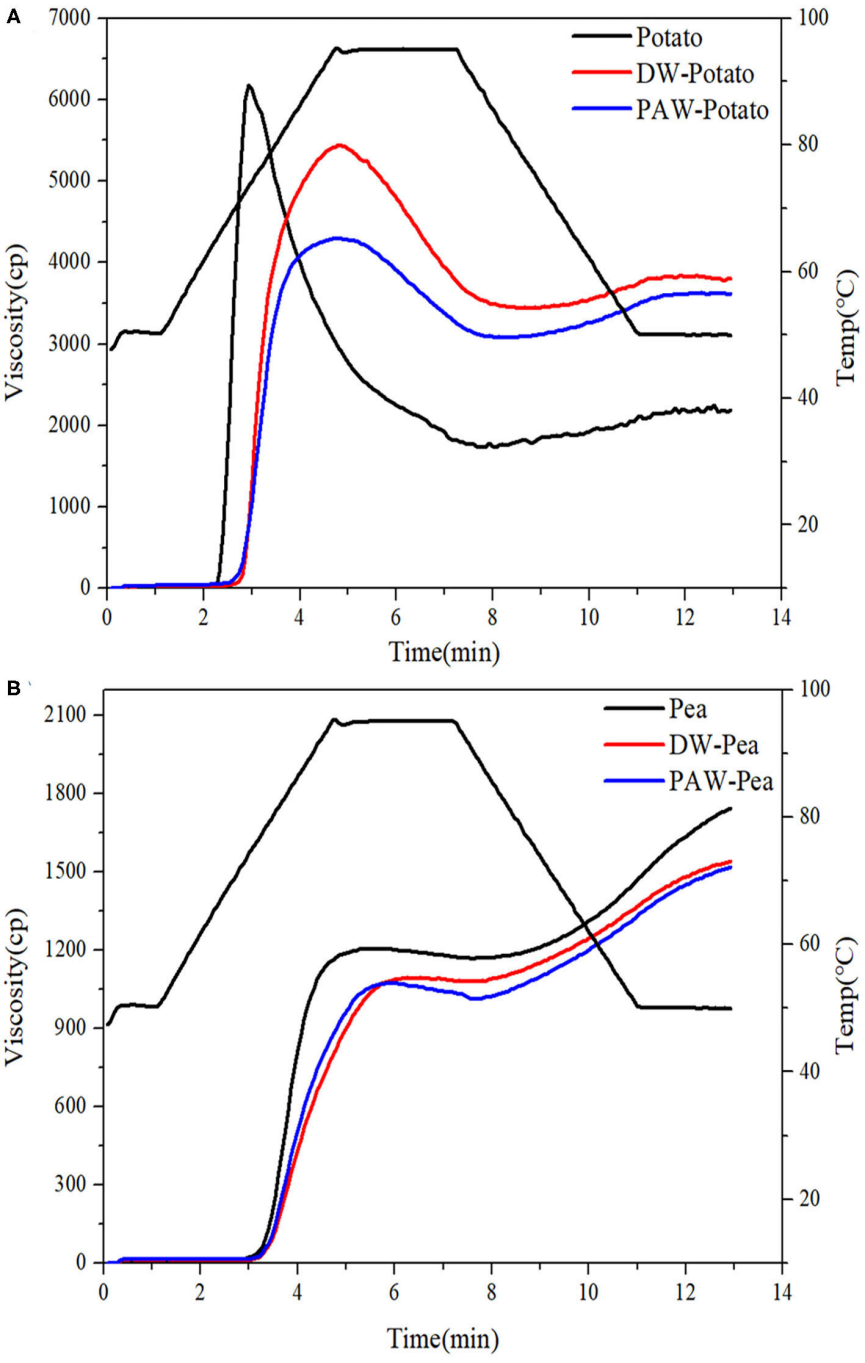
Pasting curves of native and modified starches. **(A)** Native, DW-ANN treated, and PAW-ANN treated potato starch. **(B)** Native, DW-ANN treated, and PAW-ANN treated pea starch.

The pasting characteristics curves of native and modified starches are presented in Figure 6. The viscosity change of starch granules under heating and shearing was attributed to the tightly deformed granule arrangement, the friction between the expanded granules, and the content of leached amylose and amylopectin (24). After ANN, the peak viscosity of both potato and pea starches decreased, and the peak viscosity of modified potato starch decreased more. This might be associated with a significant increase in the interaction between amylose and amylose or amylopectin, inhibiting granule swelling, which was concluded from the increase of the gelatinization temperatures of starch (25). Moreover, compared with DW-ANN, PAW-ANN resulted in a lower peak viscosity. The decrease of viscosity could be because the starch chains were largely affected by acid hydrolysis, which generated fragmentation of starch chains (26).

### Dynamic Rheological Properties

**Figure 7:**
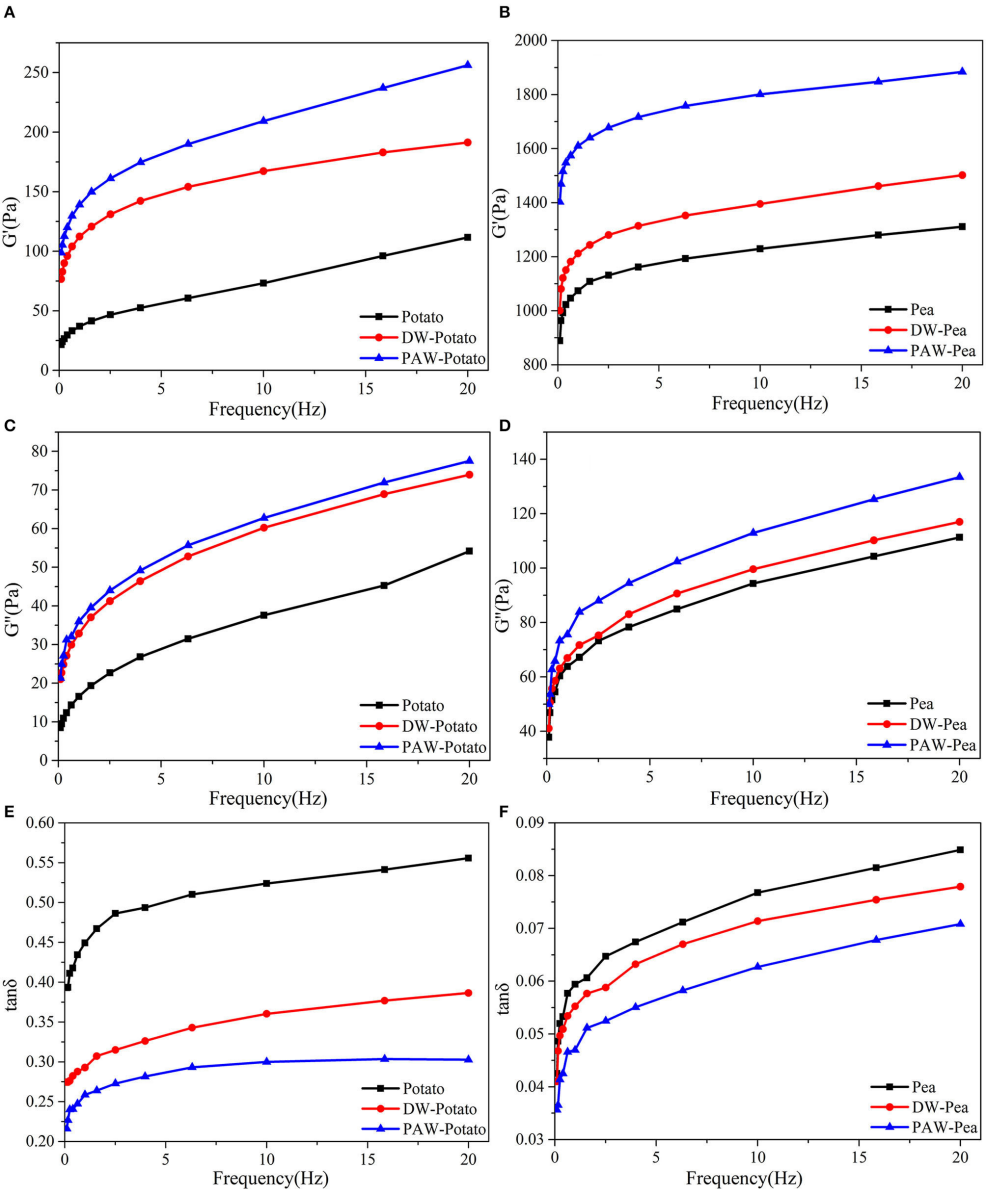
Dynamic rheological curves of native and modified starches. **(A)** Storage modulus (G′) of native, DW-ANN treated, and PAW-ANN treated potato starch. **(B)** Storage modulus (G′) of native, DW-ANN treated, and PAW-ANN treated pea starch. **(C)** Loss modulus (G″) of native, DW-ANN treated, and PAW-ANN treated potato starch. **(D)** Loss modulus (G″) of native, DW-ANN treated, and PAW-ANN treated pea starch. **(E)** loss tangent (tan δ) of native, DW-ANN treated, and PAW-ANN treated potato starch. **(F)** Loss tangent (tan δ) of native, DW-ANN treated, and PAW-ANN treated pea starch.

The dynamic rheological properties of native and modified starch pastes are displayed in Figure 7. G′ and G″ are used to represent the elasticity and viscosity of starch gels, respectively (27). Tan δ was the ratio of G″ and G′, which represents the viscoelastic properties of the starch gels (28). It can be illustrated from the Figures 7A–D that the G′ and G″ of all starch pastes increased with increasing sweeping frequency and G′ was larger than G″ (tan δ < 1), revealing that starch pastes were a typical weak gel structure and more elastic (29). After ANN, all modified starches showed higher G′ and G″, which explained that the gel strength of starch was enhanced. This may be because, during ANN, the rearrangement of amylose units formed on the surface of starch granules through larger pores and cracks increased the gel strength of starch (30). This can be demonstrated from the tan δ (Figures 7E,F). The starch pastes after ANN had a smaller tan δ, indicating that the solid properties of the modified starch pastes were enhanced with the higher gel strength. In addition, pea starch had lower tan δ than potato starch. Notably, compared with DW-ANN, PAW-ANN resulted in larger G′ and G″, and smaller tan δ, which indicated that the starch treated by PAW-ANN had higher gel strength than that treated by DW-ANN. Therefore, PAW-ANN modified starches had the highest gel strength and will be a potential gelling agent for soft candies, ice cream, and meat foods production.

## Conclusion

In summary, PAW-ANN as a completely novel starch modification method, had an obvious effect on the structure, thermal, pasting, and rheological properties of potato and pea starches. The impact of PAW-ANN on the granular morphology of two starches was not significantly different from the conventional DW-ANN. Compared with DW-ANN, PAW-ANN increased long and short-range ordered structure, and gelatinization enthalpy of pea starch while decreasing these indicators of potato starch. In addition, PAW-ANN resulted in the lowest peak viscosity and the highest gel strength of the paste for two starches. Although, the exact mechanism was not clear, an acidic component in PAW might be an important factor for starch modification according to previous studies and above experimental results. This research showed that PAW-ANN provided innovative insights and novel technologies for the production of new modified starches applied to starch-based hydrogels and food additives.

## Data Availability Statement

The original contributions presented in the study are included in the article/supplementary material, further inquiries can be directed to the corresponding author/s.

## Author Contributions

YY contributed to the conception, design, and funding of the study. BP, XJ, and YH organized the database. BP wrote the first draft of the manuscript. BN and MS contributed to writing-review and editing. All authors contributed to the article and approved the submitted version.

## Funding

We are grateful to the National Natural Science Foundation of China (32101945), the Program for Science and Technology Innovation Talents in Universities of Henan Province (20HASTIT037), and the Henan Youth Talent Support Project (2020HYTP046).

## Conflict of Interest

The authors declare that the research was conducted in the absence of any commercial or financial relationships that could be construed as a potential conflict of interest.

## Publisher's Note

All claims expressed in this article are solely those of the authors and do not necessarily represent those of their affiliated organizations, or those of the publisher, the editors and the reviewers. Any product that may be evaluated in this article, or claim that may be made by its manufacturer, is not guaranteed or endorsed by the publisher.
